# Minutes of the Mississippi Valley Dental Society

**Published:** 1876-04

**Authors:** Wm. Taft


### Proceedings of Societies.

### MINUTES OF THE MISSISSIPPI VALLEY DENTAL SOCIETY.

The thirty-second annual meeting of the Mississippi Valley Dental Society met in the lecture room of the Ohio Dental College at 10 o'clock, March 1st, 1876. The meeting was called to order and opened with prayer by the President, Dr. Geo. Watt. Reports were called for from the various standing committees, consisting of Drs. H. A. Smith, J. W. Jay, and D. W. Clancey, submitted the following subjects for discussion during the meeting, viz:

I. Dental Therapeutics.

II. Replanting extracted Teeth.

III. Sympathetic Affections caused from diseases of teeth. In what parts of the body are they most likely to occur?

IV. Filling teeth: Comparative value of different Materials and different Methods of Operating.

V. Dental Education. Do the requirements of a Dental Practice render important an acquaintance with the general principles of Medicine?

VI. Subjects connected with Mechanical Dentistry, including irregularities of the teeth.

Which was accepted and adopted.

The Committee on revising the constitution made a verbal report, the substance of which was that the Committee after considering the matter thought best not to print the old Constitution merely for the examination and suggestions by the members.

On motion Drs. J. Taft, W. H. Goddard, and H. McCullum were appointed a committee to prepare resolutions expressive of the sense of this society in reference to the death of Dr. H. R. Smith.

The hours of the sessions of this meeting were fixed as follows, viz: Morning session from 9 to 12 o'clock; afternoon session, from 2 to 5 o'clock; evening from 7½ to 10 o'clock.

On motion the Executive Committee was authorised to employ a reporter.

On motion it was ordered that all papers read before the society and the reports of its discussions be referred to the Executive and Publication Committee jointly, they having discretionary power as to the disposition of the same.

On motion the subject of "Replanting Extracted Teeth" was made the special order for 2 o'clock this p. .m, and "Dental Therapeutics" for 7½ o'clock this evening, and "Filling Teeth" for 10 o'clock a. m, to-morrow. The subject of "Mechanical Dentistry" was taken up and discussed. The Committee reported the following persons for membership in this society, viz: J. E. Cravens, Indianapolis; T. G. Dennis, Felicity, Ohio; A. B. Harryman, Aurora, Ind; J. H. Jameson, Connersville, Ind.; M. H. Chappell, Knightstown, Ind.; E. A. Igo, Rising Sun, Ind. All of whom were elected.

Adjourned to meet at 2 o'clock p. m.

### AFTERNOON SESSION.

The society met according to adjournment. the President in the chair. The subject of "Replanting Extracted Teeth" having been made the special order for this hour was now taken up for consideration. The subject was opened by the reading of a paper by Dr. W. N. Morrison of St. Louis. Dr. J. H. McKellops presented a letter from Dr. J. Hinson, of Memphis, Tenn., which made reference to his method and success in replanting extracted teeth; in this however no details were given. There was a general discussion of the subject which occupied the entire afternoon. Drs. J. Taft, Rawls, Watt, Smith, McCullum, Taylor, Leslie, Goddard and Berry taking part.

On motion a committee of three was appointed to investigate the subject of replanting teeth and report to this society at its next annual meeting. The committee consists of the following persons: W. N. Morrison, St. Louis; A. O. Rawls, Lexington, Ky.; Wm. Taft, Cincinnati.

An invitation was extended to all visitors, either dental or medical, who may be present to participate in the discussions.

Adjourned to meet at $7\frac{1}{2}$ p. m.

### EVENING SESSION.

The society met according to adjournment. The discussion upon the subject of the afternoon was continued for an hour; after which, the first subject on the programme, "Dental Therapeutics" was taken up and discussed till adjournment.

### SECOND DAY, MORNING SESSION.

The Society met at the appointed hour. Filling teeth was the subject in order for consideration. The subject was introduced by Dr. McKellops; he presented a number of new appliances, among which was the diamond drill, the hard rubber disks and cones; the method of making and the manner of using were fully explained by the Doctor. A vote of thanks was passed for the exhibition and description of these appliances. Adjourned.

(If the members of our societies generally would manifest the same interest and effort, usually shown by Dr. McKellops, our meetings would be vastly more interesting and instructive. Ed.)

### AFTERNOON SESSION.

The subject of filling teeth was resumed. Upon invitation Dr. Jas. Leslie gave a demonstration with his crystalline gold, after which the discussion of the subject was closed, and Mechanical Dentistry was taken up, more particularly with reference to the appliances for correcting irregularities of the teeth. Remarks were made by Drs. McKellops, G. W. Keeley and C. R. Taft. The latter two gentlemen presented interesting models in illustration of the views they advanced.

The election of officers was now declared to be the next order of business, which being done the following were found to be elected for the ensuing year, viz:

President, J. H. McKellops.

1st Vice-President, J. S. Cassidy.

2d Vice-President, A. O. Rawls.

Recording Secretary, Wm. Taft.

Corresponding Secretary, A. B. Harryman.

The President elect took his seat, and the retiring President delivered his address.

The following were appointed delegates to the American Dental Association: J. R. Clayton, M. H. Chappell, J. E. Cravens, C. P. Dennis, J. I. Taylor, F. W. Sage, C. R. Taft, H. McCullum, D. W. Clancey, W. Storer Howe, R. A. Molyneux, S. E. Harryman, P. Meredith, Jas. Leslie. The delegation was empowered to fill vacancies. Adjourned.

THIRD DAY, MORNING SESSION.

The discussion upon Mechanical Dentistry was continued.

Dr. J. W. Baxter, of Vevay, Ind., exhibited a new and improved impression cup, describing its use. He presented it to the profession free of any restrictions by patent or otherwise.

The following standing Committees were appointed:

| | |
|---|---|
| ETHICS. | Geo. W. Keeley, Oxford, O.<br>H. M. Reid, Cincinnati, O.<br>W. N. Morrison, St. Louis, Mo. |
| MEMBERSHIP. | S. B. Brown, Ft. Wayne, Ind.<br>A. L. Emminger, Columbus, O.<br>J. E. Cravens, Indianapolis, Ind. |
| APPLIANCES. | W. H. Goddard, Louisville, Ky.<br>J. R. Clayton, Shelbyville, Ind.<br>J. W. Baxter, Vevay, Ind. |
| EXECUTIVE. | C. R. Taft, Cincinnati, O.<br>F. A. Hunter, Cincinnati, O.<br>H. A. Downing, Cincinnati, O |

PUBLICATION. { Geo. Watt, Xenia, O.

H. A. Smith, Cincinnati, O.

Wm. VanAntwerp, Mt. Sterling, Ky. }

The Committee appointed to draft resolutions in reference to the death of Dr. H. R. Smith, presented the following:

WHEREAS, By the dispensation of an all-wise Providence we are called upon to record the death of one of our members within the past year; it becomes us to bow in uncomplaining submission to the behests of infinite wisdom, as exhibited in this additional intimation of our own mortality, and while we breath a sigh to the memory of our departed friend and tender our sympathy and condolence to his family, we treasure the memory of his many virtues, as valued memorials of our pleasant associations here, and his example as worthy of our imitation. Genial and aimable in his social character, he never hesitated to express his convictions of truth in the most inequivocal and emphatic terms. Therefore,

*Resolved*, That in view of the loss our profession has sustained in the demise of our friend, brother and co-laborer, we deem it proper that we give expression to our high appreciation of his personal, social and professional worth; his untiring zeal for the advancement of our profession; his unswerving friendship for those engaged in kindred pursuits, these qualities commend his character to a place in the memory of all who knew him, and especially to the members of this Society.

Tendering our united sympathies to the members of his bereaved family, we feel it incumbent upon us to address ourselves with renewed energy to the discharge of our individual and relative duties remembering that with each and all of us time is short.

{ W. H. GODDARD,
H. MCCULLUM,
J. TAFT. }

Dr. J. Taft was appointed a committee to correspond with the Committee of Arrangements of the American Dental Association with the view of securing for the dental profession ample accommodation during the Centennial Exposition and especially during the time of the meeting of the American Dental Association, and to report through the journals at the earliest practicable period.

Adjourned to the next annual meeting.

WM. TAFT, Sec'y

